# Rheological Behavior and Printability Study of Tri-Calcium Phosphate Ceramic Inks for Direct Ink Writing Method

**DOI:** 10.3390/polym15061433

**Published:** 2023-03-14

**Authors:** Belgin Paul D L, Ayyappan Susila Praveen, Lenka Čepová, Muniyandy Elangovan

**Affiliations:** 1 Department of Mechanical Engineering, Vel Tech Rangarajan Dr. Sagunthala R&D Institute of Science and Technology, Avadi 600 062, India; draspraveen@veltech.edu.in; 2 Department of Machining, Assembly and Engineering Metrology, Faculty of Mechanical Engineering, VSB-Technical University of Ostrava, 17. Listopadu 2172/15, 708 00 Ostrava, Czech Republic; lenka.cepova@vsb.cz; 3 Department of R&D, Bond Marine Consultancy, London EC1V 2NX, UK

**Keywords:** additive manufacturing, tricalcium phosphate, rheology, direct ink writing, scaffold, dimensional error, compressive strength

## Abstract

In the biomedical industry, tricalcium phosphate is a bioceramic substance that is frequently employed in the fabrication of scaffolds and bone structures. Fabrication of porous ceramic structures using conventional manufacturing techniques is very challenging because of the brittle nature of the ceramics, which has led to a newly adapted direct ink writing additive manufacturing method. This work investigates the rheology and extrudability of TCP inks to produce near-net-shape structures. Viscosity and extrudability tests found that stable TCP: Pluronic ink of 50 vol.% was more reliable compared to other tested inks prepared from a functional polymer group polyvinyl alcohol. A line study was carried out to identify the printing parameters suitable for printing structures from the selected ink with lesser dimensional error. Printing speed 5 mm/s and extrusion pressure 3 bar was found suitable to print a scaffold through a nozzle of 0.6 mm, keeping the stand-off distance equal to the nozzle diameter. The printed scaffold was further investigated for its physical and morphological structure of the green body. A suitable drying behavior was studied to remove the green body without cracking and wrapping before the sintering of the scaffold.

## 1. Introduction

Over the past forty years, additive manufacturing (AM) has undergone significant growth due to its advanced manufacturing benefits over the conventional subtractive method. AM can be utilized with a variety of materials, including ceramics, metals, and plastics [1,2,3,4]. Among these, the manufacturing of ceramics and metals using AM is being investigated by the research community due to its engineering applications. AM is used widely in many industries such as automotive, aerospace, biomedical, electronics, and more [5,6,7,8]. Due to their fragility, high melting temperature, and limited ductility, ceramic structures are the most difficult to manufacture compared to metals, making AM an essential tool. There are many AM techniques used for processing ceramics including “selective laser sintering (SLS) [9,10], stereolithography (SLA) [11,12], direct ink writing (DIW) [13,14], Binder jetting [15,16]”, etc. Though all the abovementioned methods are reliable for AM of ceramics, DIW is considered to be the most effortless and cheapest method for the production of ceramic structures [17,18]. 

Among the available ceramic materials, tricalcium phosphate (TCP) has implicit great capability for its biocompatible nature, which opens up endless applications in the biomedical field [19,20]. DIW of tricalcium phosphate is in the spotlight nowadays for the production of scaffolds for in vitro studies and bio-implants for in vivo application in the biomedical field [21]. A scaffold needs to have micro- and macro-pores internally to achieve rapid and stable cell growth. Through the adaptive DIW methods, those pores can be made at the required size, which may replicate a natural bone structure [22]. The strength of the prepared scaffold can be increased or decreased based on the percentage of solid loading, mixing other materials with the base material, and altering the infill density of the scaffolds. The rheology of the ink used for the DIW technique has a greater significance in the stability and fragility of the 3D printed structures. Therefore, understanding the basic parameters that influence rheology must be studied. Viscosity (µ), shear stress (τ), shear strain (Υ), storage modulus (G′), and loss modulus (G″) are a few important properties to be considered in the rheology of the prepared ink. These properties determine the ink that is suitable for printing perfect 3D printed structures. D. Korachkin et al. briefly explained the change in rheological properties with a change in the volume fraction of the powder [23]. T. J. Carneim and D. J. Green have pointed out the effect of storage and loss moduli of green bodies prepared from alumina powder [24]. N. Somers et al. made a slurry for DIW from doped and un-doped TCP and investigated the compressive strength and density. The characterization study demonstrated that the doped TCP had reduced pore size and significantly decreased risk of crack formation [25]. H. Zhu et al. studied the usage of TCP cement mixed with pluronic and its setting, as well as the storage time of the paste prepared [26]. The study on prepared TCP structures was also found to be the most useful for clinical applications. J. Zhang looked into how the sintering temperature affected the size of the pores in the scaffolds made from TCP and pluronic gel. It was observed that the pore sizes are macro-pores and decrease in size with the increase in sintering temperature. An in vitro clinical study demonstrated promising bioactivity in the scaffolds [27]. Zhimin Xu and others developed scaffolds from β-TCP/PVA/dipyridamole composite using 3D printing. To measure the cells’ osteogenic activity, the scaffolds were seeded for drug delivery. There was no change in the strength of the scaffold after new cells were grown into the structure. Therefore, the scaffolds developed were concluded to be more bioactive, with cell proliferation abilities [28]. Though previous studies have shown TCP as a material for scaffolds with active bone regeneration, the influence of the TCP ink’s rheological characteristics on extrudability, dimensional stability, and drying behavior through the DIW process has not been studied in any systematic way. 

This manuscript aims to provide guidelines for producing TCP ink suitable for the DIW method by studying the rheological properties of the inks prepared from pluronic and PVA in different proportions. Additionally, the best ink suitable to extrude continuously was selected from an extrudability test and line study through a 0.6 mm diameter nozzle. Further, a scaffold was printed to evaluate the compressive strength, dimensional error, pore size, and drying behavior without any crack formation. Overall, this work gives insight into the flow behavior of TCP ink through a nozzle to identify the safe printing of scaffolds for engineering and biomedical applications.

## 2. Experimental

### 2.1. The Feedstock of the Ink

The tricalcium phosphate powder (99.5% β-Ca_3_(PO_4_)_2_, Sisco Research Laboratories, Mumbai, India., average molecular weight 310.18 g/mol,) was mixed thoroughly with a deionized water-based solution made from two binders, polyvinyl alcohol (PVA) (99.2% C_2_H_4_O) X, Sisco Research Laboratories, Mumbai, India., average molecular weight 44.05 g/mol) and pluronic (99% pluronic F-127, Sigma-Aldrich, Bangalore, India, average molecular weight 12,600 g/mol). These materials and binders were selected because of their biocompatibility and their being commonly used in DIW. The inks prepared from TCP-PVA and TCP-pluronic F-127 were studied for investigating the suitable vol.% with the binders PVA and TCP. Moreover, the effects on rheological behavior, their binding, and dispersive properties were studied. PVA powder was dissolved in deionized water and stirred with a magnetic stirrer at 60 °C for one hour to create PVA solution. The temperature was maintained at 60 °C because water starts to evaporate at higher temperatures ranging from 90–100 °C. However, pluronic F-127 does not require heating because of its special photo-thermal properties. To prepare the pluronic gel, the power pluronic F-127 was mixed with deionized water and the solution was kept in a freezer below 4 °C for 24 h. The solution obtained was a homogeneous solution that becomes a hydrogel when kept at room temperature. These PVA binders and pluronic gel were then mixed with TCP powder using a mechanical stirrer for 1 h to follow specific vol.%, which was named as sample names A:B (for example 50:50 PVA), where A is the TCP ratio and B is the ratio of PVA. Three different groups of ink were prepared from the selected TCP solid loading and the binder or hydrogel (i.e., 50, 55, and 60 vol.%). The ink thus prepared was then put through a viscosity test and printability checks for the DIW technique.

### 2.2. TCP Characterization and Rheological Tests of the Ink

Particle size distribution of TCP powder was conducted on a Mastersizer 3000E (Malvern Panalytical Ltd., Malvern, UK) and morphology analysis on a “scanning electron microscope (SEM) (Tescan Vega 3)” using a sample pinch of powder with a specific surface area of 1408 m^2^/kg and particle refractive index of 1.620. Rheology tests were performed on the rotational rheometer Bohlin Gemini II (Malvern Panalytical Ltd.) using two parallel plates with a 1 mm gap. The tests were performed at 25 °C ranging from 100 to 0.01 s^−1^.

### 2.3. Extrudability

In the present work, the prepared TCP ink was extruded through a 0.6 mm cone-type nozzle attached to the barrel. The ability of the ink to extrude out continuously upon pressure and capable of print continuous lines were categorized using the following criteria: (a) un-extrudable, (b) uncontrollably extrudable, (c) inconsistently extrudable, (d) controllably extrudable. A successful ink should be able to extrude controllably and continuously.

Extrudability tests were carried out using line printing and scaffold printing. In line printing, lines were printed through the 0.6 mm nozzle to check their line width and continuous printing pattern, whereas in scaffold printing, a scaffold was printed with dimensions 20 × 20 × 5 mm. This scaffold printing was performed to check the feasibility of printing larger-dimension objects by considering only the controllably extrudable and inconsistent extrudable inks, eliminating the former two criteria.

DIW printing was carried out using a 3D printer (Tevo Tarantula, Odisha, India) that was locally customized and coupled to an air compressor with a maximum output of 7 bar. The head was changed out for a dispensing barrel with a capacity of 30 cc and a 0.6 mm cone-type nozzle.

## 3. Results and Discussion

### 3.1. Particle Size and Rheological Evaluation

**Figure 1:**
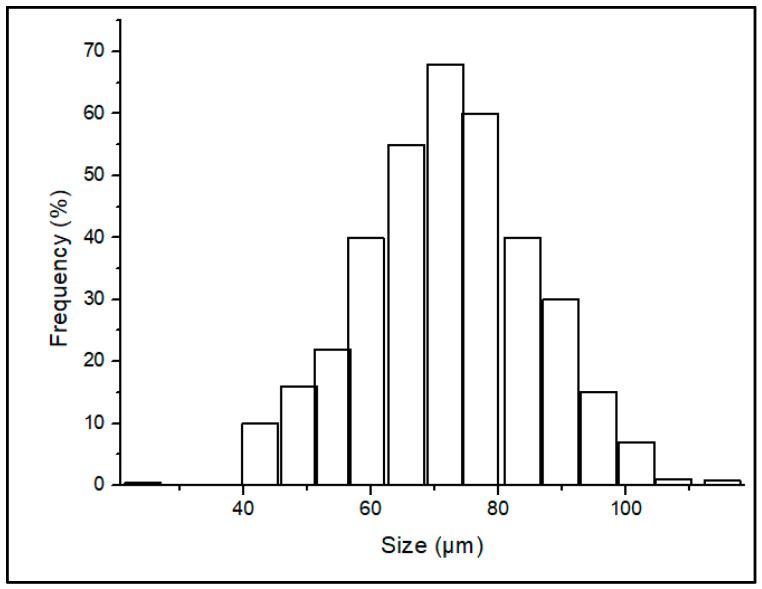
Particle size distribution of the TCP feedstock.

A wide distribution was found in the TCP powder analyzed under the laser particle analyzer given in Figure 1. The software used was mastersizer–v3.62 and the average size distribution was 5.592 µm at a specific surface area of 1413 m^2^/kg.

Using a rheometer, the viscosity (µ) property of the produced inks was assessed to ascertain the shear thinning behavior. An important requirement for DIW ink to satisfy is effective printing without any clogs or uncontrollable extrusion in the nozzle. When prepared with only water, inks have unstable viscosity and it is difficult to predict the flow through the nozzle. There may be possible agglomeration, which may increase the risk of clogging at the nozzle tip [29]. To overcome this randomness in rheology, polymers and hydrogels such as PVA and Pluronic F-127 were added with the TCP powder to make it into ink, which may flow with certain rheological behavior. Based on the trial experimental works, it was discovered that to print a structure with full density capable of sintering, it must be over 50% and less than 60% [30]. To comprehend the rheological characteristics of the ceramic ink, it is crucial to grasp the storage modulus (G′) and loss modulus (G″) key parameters. A Malvern-Bohlin Gemini II cone and plate type rheometer was used in the rheological investigation, which was conducted at various shear rates and frequencies. The G′ and G″ of the ceramic ink utilized for the DIW method were determined using the complex modulus (G*) method. G″ is the capability of the prepared ceramic ink to dissipate the stored energy when a force is applied, whereas G′ is the capacity of the prepared ceramic ink to retain energy.

**Figure 2:**
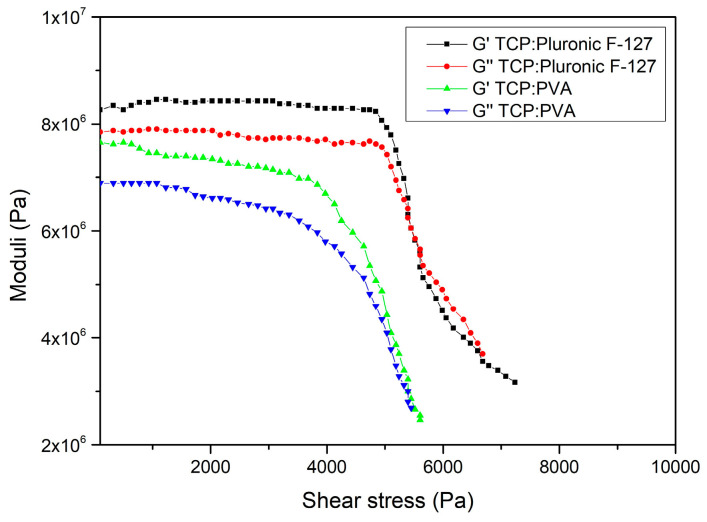
Effect of storage and loss moduli on rheology of the ceramic ink.

Initially, G* was calculated by dividing stress by strain. It was then multiplied with cos(δ) to obtain G′ and G″ was obtained by multiplying the complex modulus with sin(δ), where δ is the phase angle, usually 90° for a viscous fluid. When G′ > G″, the ink tends to follow shear thinning behavior and the resistance offered by the storage modulus will be overcome by the shear force and the ink flows. Figure 2 interprets the behavior of the storage and loss moduli with the increase in shear stress on the prepared ceramic inks.

Apart from storage modulus, other properties contribute to the rheology of the inks, which affects the printability of the ceramic inks. These are referred to in the Herschel–Bulkely model stated below in Equation (1):

(1) $$\tau =\tau_{y}+k\;\times \;{\left(\gamma ˙\right)}^{n}$$

where (τ) is the applied shear stress to the ceramic ink, yield stress ($\tau_{y}$) denotes the minimum shear stress needed for plastic deformation, (*k*) is the viscosity parameter, (*γ*˙) is the shear strain, and (*n*) is the shear thinning coefficient. This equation has been identified as very much important in representing the flow of inks used in DIW. For an ideal ink, $\tau_{y}$ > 0 and *n* < 1 indicate the shear thinning phenomenon, where the resistance to flow decreases with an increase in shear rate. The inks should behave in this way to provide print stability to the structures and stop the flow whenever and wherever required per the design model. The other behaviors are Newtonian fluids (*n* = 1 and $\tau_{y}$ = 0) and shear thickening (*n* > 1), which means µ increases with the shear rate. The ceramic powder used as solid loading perhaps has an effect on the rheology of the ink with the volume fraction and surface chemistry. The grain size should be approximately 50 times smaller than the size of the nozzle used and the volume fraction should be in a nominal range to prevent the dilatant effect, which clogs the nozzle. Both the prepared inks from the pluronic gel and PVA exhibited a viscoelastic region when the stress was low until yield stress. The surface chemistry of the inks demonstrated inter-particle interaction of the particle and the dispersants.

**Figure 3:**
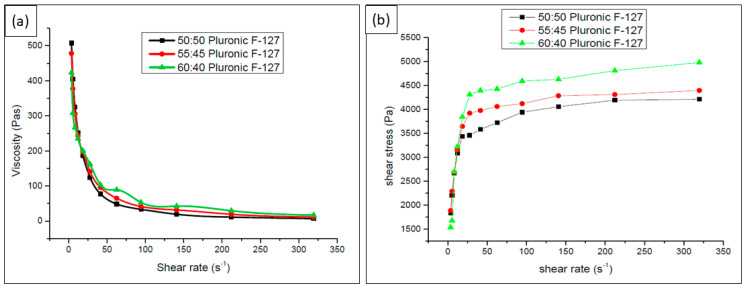
Viscosity property on TCP: Pluronic F-127 ink with different volume ratios. (**a**) Shear rate vs. viscosity and (**b**) shear rate vs. shear stress.

In the range of 22 to 100 s^−1^, pluronic F-127 added to TCP powder demonstrated shear thinning behavior, as depicted in Figure 3a through the modification of shear viscosity. Meanwhile, in other ranges, it demonstrated shear thickening behavior. This is because of the intermolecular attraction of the suspended particle in the ink, which leads to agglomeration at a certain value of shear rate. When this phenomenon increased, the clogging rate at the DIW printer also escalated. It was also found that when no pressure is applied, the storage modulus (G′) was greater than the loss modulus (G″) and thus behaved viscoelastically in nature, with G′ equal to 10 MPa. It flowed easily upon the shear force with G″ > G′. From Figure 3b, it is observed that the elastic reaction occurs at the beginning and then the ink follows a shear-thinning behavior. Furthermore, between the range of 0–22 s^−1^, there is a fluctuation in the curve before the ink starts to stabilize on the logarithmic increment. The increase in shear rate from 3500–4500 Pa shows some stabilization in the flow behavior and thus can be extruded continuously after a steady state occurs. Therefore, the prepared ink can be classified as controllably extrudable.

**Figure 4:**
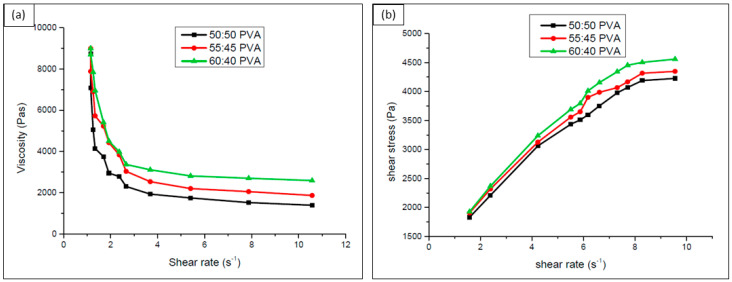
Viscosity property on TCP: PVA ink with different volume ratios. (**a**) Shear rate vs. viscosity and (**b**) shear rate vs. shear stress.

The ink samples prepared from various volume ratios of PVA to the TCP powder display a decrease in viscosity to the shear rate, as shown in Figure 4a. The curves moving down with the increase in shear rate present clear evidence of the ink changing its phase from shear thickening to shear thinning from the range 1 to 4 s^−1^. In addition, the storage modulus decreases from 8 to 1 Mpa from the initial value while breaking down from viscoelastic to shear thinning behavior (G″ > G′). This is because of the binding property of the PVA to the loaded TCP particles, which forms ionic interactions with the functional groups of PVA. The inks also stabilized in flow after certain extrusion pressure was applied as shear stress increased concerning the shear rate as presented in Figure 4b.

### 3.2. Extrudabitity Test

**Figure 5:**
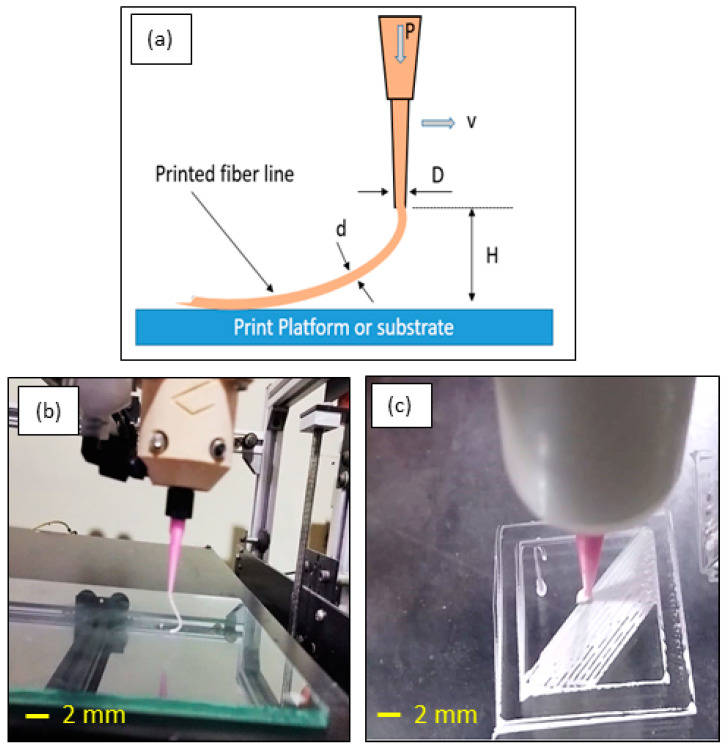
(**a**) Schematic of extrudability, (**b**) controllably extrudable, and (**c**) die-swelling.

Among the various inks discussed in the rheology study, only the inks prepared from 50, 55, and 55 vol.% TCP: Pluronic were able to extrude out through the 0.6 mm nozzle at a pressure range of 3–6 bar. No die-swelling was observed when extruded through the nozzle. Die swelling is a tendency for ink to expand at the exit of the nozzle, making it unstable in the dimensional property. Figure 5 shows a schematic representation of die swelling and video stills of the inks with and without the die swelling effect. In the diagram, P stands for the applied pressure, D for the nozzle’s tip diameter, d for the fiber line’s wire diameter, v for the head’s printing or transverse speed, and H for the standoff distance between the nozzle tip and the platform. Therefore, d > D near the exit of the nozzle is the necessary condition for die-swelling. Thus, it was noted that the prepared pluronic-based inks are controllably extrudable. On the other hand, the inks prepared from PVA were observed to be inconsistent and extrudable. The inks from 50, 55, and 60 vol.% TCP: PVA could not be printed continuously in the above-said pressure range and demonstrated blockages at the nozzle head, which caused a die-swelling effect at some time and thus a lack of predictability. However, increasing the nozzle size to 0.8 mm and above demonstrated satisfactory extrusion, although that compromised the printing resolution. In addition, PVA inks have the negative property of being converted into a thick paste over time [29].

### 3.3. Line Study

**Figure 6:**
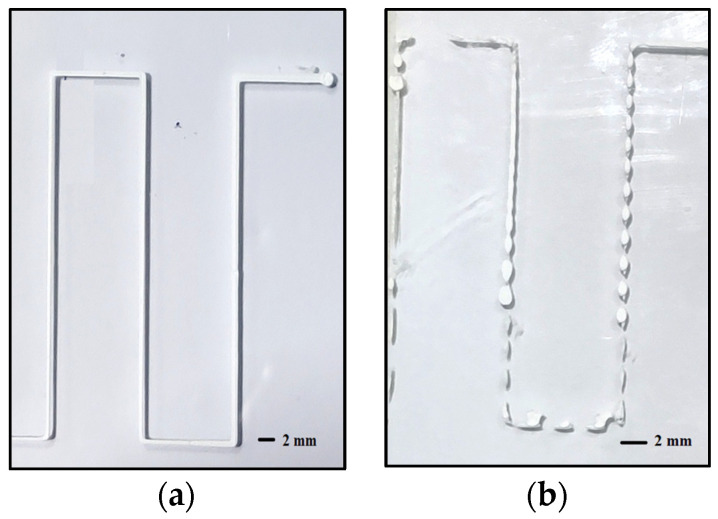
Sample line study of (**a**) controllably extrudable and (**b**) inconsistent extrudable inks.

A line study is performed on the prepared inks to identify the printability and the behavior of the viscoelastic inks in detail. Lines are printed on a substrate with standoff distance H = 0.6 mm (diameter of the nozzle) and varying the print speed (5 mm/s–10 mm/s) and pressure (3–6 bar). The line study evidenced that the ink made of 50 vol.% TCP: pluronic, p = 3 bar, v = 5 mm/s printed nearly equidimensional lines, as shown in Figure 6a, whereas the other inks demonstrated various other possibilities such as accumulation of the inks, coiling, die-swelling, thinning and discontinuous lines, as shown in Figure 6b.

### 3.4. DIW Printing and Evaluation of Scaffold

**Figure 7:**
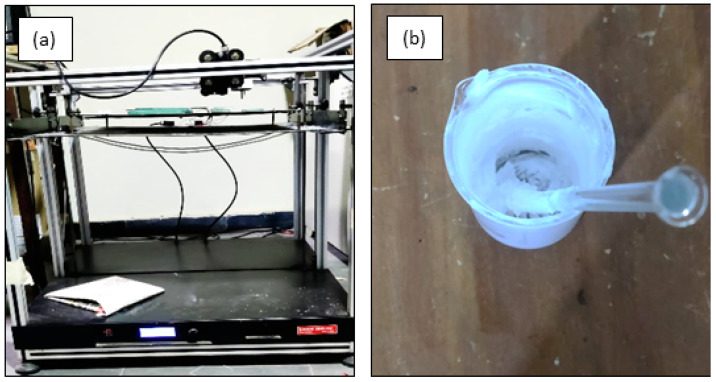
(**a**) DIW printer used in the study. (**b**) Prepared pluronic-based TCP ink.

**Figure 8:**
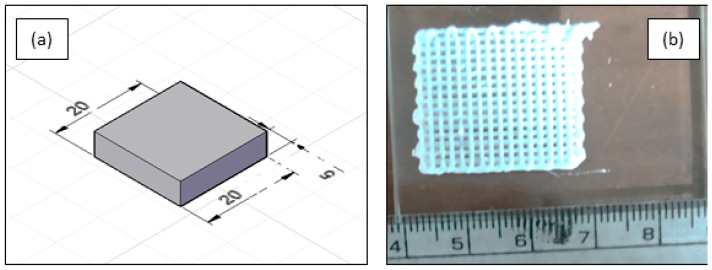
(**a**) Three-dimensional CAD model of the scaffold. (**b**) DIW Printed scaffold.

By evaluating the combined results from the rheological test, extrudability test, and line study, the pluronic-based ink of 50 vol.% of solid loading was found to be the suitable ink to print near-net-shape structures using the DIW method. This proposition not only shows a shear thinning behavior but also provides continuously extrudable inks through the nozzle without die-swelling. With that being said, the process parameters identified were used to print scaffolds of a square cross-section to identify the quantitative feasibility of a reproducible structure. The DIW printer and the ink used in the current investigation to print scaffolds are shown in Figure 7a,b. The CAD model of the scaffold of dimension 20 × 20 × 5 mm was made using fusion 360 software as shown in Figure 8a. The designed model was sliced using Cura software to slice and generate G-codes, which were given as input to the DIW printer. The printed scaffolds in Figure 8b with a 60% infill rate were evaluated for their morphology, dimensional stability, porosity, and compressive strength.

**Figure 9:**
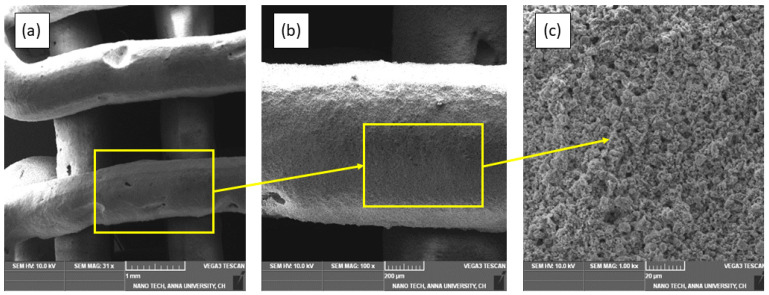
Morphology of the printed scaffold. (**a**) SEM image showing line fibers printed over each other. (**b**) SEM of a single line strand. (**c**) Magnified image of the particles of the line.

Using a scanning electron microscope (SEM, TESCAN VEGA 3), the printed scaffold was examined for the morphology of the printed lines and the binding of one layer over the other. Figure 9a provides a clear sign of the adhesion of layers over one another about the circular shape of the line strands without any spreading and distortion. This occurrence is because of the vector nature of the identified ink and its shear-thinning behavior. The print speed of 5 mm/s used in this printing process was also found to be fine-tuned because of the continuous curve at the edges without any discontinuity. In addition, there were many voids found in the strands as highlighted, which are because of the degradation of the hydrogel and the air voids in the prepared ink. Therefore, it is necessary to mix the ink thoroughly to produce a homogeneous ink without any air voids. The line strand and the printed line strand particles are depicted in magnified SEM images in Figure 9b,c, respectively. It can be seen that the particles are distributed homogeneously and there is good bonding between the particles. This helps the printed structure be more strong and less brittle, making it residual-powder-free after printing. It is also evident from the SEM image that the particles homogeneously formed a link with the hydrogels to form a solid structure. Therefore, any additional reagents were not required for the observed green sample. The pore size of the scaffold measured at five pores was found to be over 100 μm. This is because of the infill density of 60%, which was selected to print macroporous structures. Macroporous structures are necessary for a scaffold to be used in cell culture and osteointegration [31].

**Figure 10:**
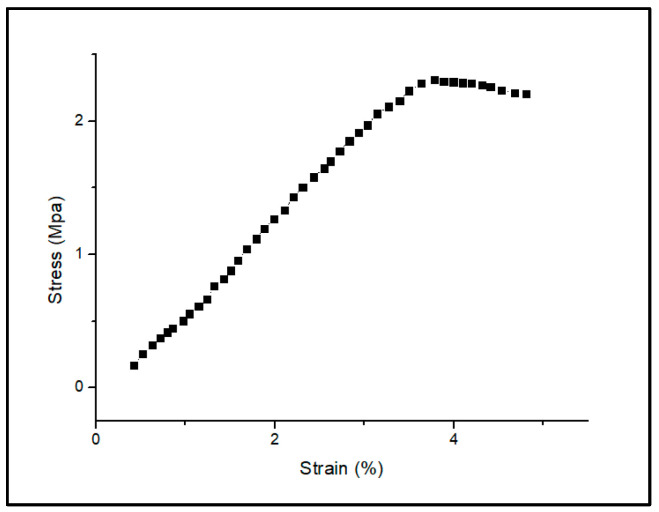
Compression test showing stress vs. strain of the printed scaffold.

By measuring the length, breadth, and height of the prepared scaffold and comparing them to the original measurements of the CAD model, the dimensional stability of the scaffold was examined. The average deviation from the original dimension is referred to as dimensional error in percentage. The lesser the dimensional error, the more the dimensional accuracy of the printed structure. The dimensional error was calculated using the formal given in Equation (1)

(2) $$\mathrm{Dimensional}\;\mathrm{error}\;(\%)=\frac{\left(D_{a}-D_{m}\right)}{D_{m}}\;\times \;100$$

where actual size is measured from the printed scaffold and dimensions from the CAD model. The printed green sample’s average dimensional inaccuracy was determined to be 1.62%, which is lower than the other production processes mentioned in the literature [32]. Using a universal testing device, the scaffold’s compressive strength was evaluated (WDW-100) and the compressive strength of the green sample was measured as 2.31 MPa as shown in Figure 10, which is nearly the same as in other works carried out in the literature [33]. Similar works reporting compressive strength and porosity with similar inks and various other processes are given in Table 1.

**Figure 11:**
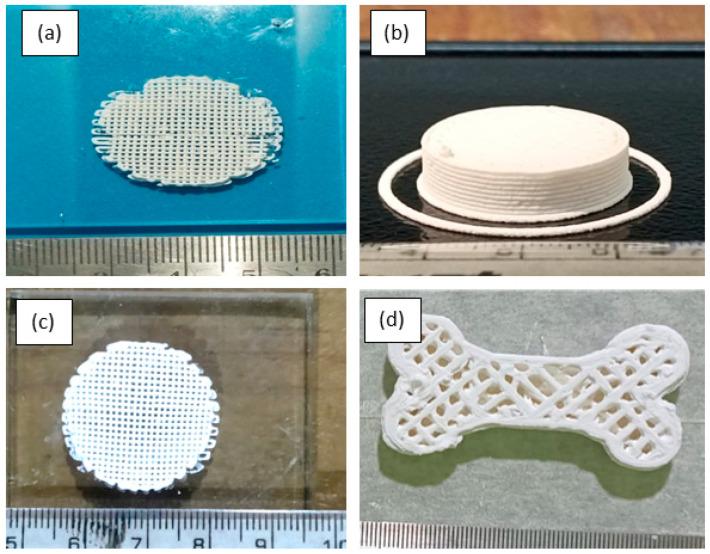
Samples printed with larger part size. (**a**–**c**) Circular scaffold. (**d**) Bone structure.

To find the feasibility of printing with a larger part size, a circular scaffold and a cross-section of a bone were printed as shown in Figure 11a–d. It was seen that when larger size structures are printed, there may be some air voids coming out during the flow process at the end when the ink was about to exhaust. This may be avoided by adding suitable additives in smaller vol.% in addition to the hydrogel, and the mixing process can be carried out in a vacuum mixer for about 1 h after the usual mixing with a mechanical stirrer.

### 3.5. Drying Behavior

The green sample body has to be studied for its drying behavior because of its impact on the dimensional stability of the result due to shrinkage [36]. This happens when a solvent or a binder is removed from the printed green bodies. Therefore, the drying process should be even, otherwise the printed structure may dry to an uneven shape with irregular dimension and cause wrapping [37,38]. Usually, the ceramic structures printed by the DIW method often produce cracks when detached from the print substrate. To eliminate this, greasy gel was used on the surface of the substrate before printing. After detaching from the substrate, the scaffold was allowed to undergo a drying process in a low-humidity atmosphere. The green sample was initially weighed immediately after printing and then kept at a room temperature of 30 °C for 4 h, then kept in a furnace and maintained at a temperature of 60 °C for 2 h. The temperature was then gradually raised to 150 °C and maintained at that range for 1 h. It was observed that the weight loss ratio was 2.36% because of the decomposition of gas molecules and evaporation of water content in the sample. Further, the temperature was increased to 350 °C, and as the pores were open because of the entrapped gas inside the line strands, the weight loss ratio was measured as 0.96%. The temperature was increased further to 500 °C and the weight loss was measured to be 0.39%, with no further considerable change in the weight of the sample thereafter. This shows that the organic matter present in the printed sample was completely wiped out. 

## 4. Conclusions

Various contrasting ink compositions were prepared from TCP powder and tested for their rheology to identify the feasibility of inks for the DIW method. A stable TCP: Pluronic ink of 50 vol.% was identified from the systematic investigation of various TCP inks, giving an extensive collection of parameters for DIW. The proposed ink was found to be suitable for the extrusion process due to its shear thinning behavior under shear force. It also provides good controllably extrudable ink unlike the other different inks prepared from PVA, which exhibits inconsistently extrudable behavior. Printing process parameters identified from extrudability and line study were 0.6 mm nozzle diameter, 5 mm/s print speed, 0.6 mm standoff distance, and 3 bar pressure. A printed porous scaffold was tested for its dimensional stability, morphology, compressive strength, and drying behavior. The green sample had an acceptable dimensional error, compressive strength, and macro pores, which are suitable for cell growth for biomedical applications. Moreover, a drying sequence was studied to prepare the green sample before the sintering process, which is not in our current scope of work.

## Figures and Tables

**Table 1:** Porosity and compressive strength of scaffolds in the reported studies.

Process	Porosity (%)	Compressive Strength (MPa)	Reference
DLP	74	4.09	[24]
Gel casting	73	6.8	[31]
DIW	92	32	[34]
Die casting	73	1.63	[35]
DIW	84	2.31	Current work

## Data Availability

The data presented in this study are available through email upon request to the corresponding author.
